# Doctors and Patients as Artists See Them

**Published:** 1920-01-31

**Authors:** 


### THE NATION'S WAR PICTURES AT BURLINGTON HOUSE.

### Doctors and Patients as Artists See Them.

Throughout December and January the works of art belonging to the Imperial War Museum have been on exhibition at Burlington House. Two things must have occurred to those visitors who are familiar with many of the sights and scenes represented in the galleries: one, the astonishing ignorance of those who stayed at home of what the Great War was really like, in spite of the efforts of journalists and special war artists to tell them; and the other, the astonishing failure of most of the pictures to tell the plain truth to these uninstructed people. Possibly the two things are more closely connected than one had previously realised. The veriest commonplaces of the trenches still seem unbelievable, apparently, to those who have not seen them, which makes it all the greater pity that so many of the pictures about those ditches of death should be unfaithful to the reality. Frankly, the Committee would have been better advised to hang about a quarter of the exhibits, and even then there would have been many indifferent ones admitted to the exhibition. The failure of cubist, impressionist, neo-experimentist schools of art in general, to tell the plain truth about the horrors of war—a subject specially suitable, one might have thought, to blunt and uncompromising realism—is painfully apparent in many of the daubs which misrepresent man and nature in this exhibition.

### Great Interest and Artistic Value.

Premising, then, that expectations about the Nation's war pictures are not to be pitched too high, there is yet much of great interest and of artistic value in the exhibition. The pictures and drawings of dressing-stations and hospitals are, like the rest of the collection, of very varying merit. Mr. Sargent has devoted some of his genius to medical topics: his great canvas "Gassed," which was one of the pictures of the year at the last Academy, is not only magnificent, but is also war. Another painting (452) by this artist of a hospital marquee is one of the gems of the collection, both for sincerity and colouring; it is quite the best of his smaller canvases.

Professor Tonks, F.R.C.S., of the Slade School, shows a good many pictures, some of them medical in subject and many of them admirable. Of his hospital pictures, that of an advanced dressing-station is really good (77), though on a similar theme (20) he runs it close. A picture of saline infusion at a Red Cross hospital (429) is not nearly so good, and has a scamped appearance.

Sir William Orpen, who exhibits a great many pictures, has not devoted himself to medical topics: some of his portraits are first-rate, but he does not always keep to his highest levels. It is fair to add that many of his war pictures have not yet returned from America; and no doubt many of his very best ones were sent to represent British art there. His portrait of Field-Marshal Lord Plumer is splendid; but to the latter's gallant Second Army at large their G.O.C. will always be associated with a "coat, warm British" and a characteristic figure and gait: all this is lost in a half-length indoors portrait of him, which can never be quite the Plumer they knew best.

### Some Contrasts.

Some of Mr. Nevinson's pictures are marvellous; at his best he can bear comparison with any one (113 and 238). At his worst he is dreadful, and unfortunately "The Doctor" (246) is in the second category: it strikes one as fundamentally dishonest, though in view of his long association with the R.A.M.C. it is more probably merely a blunder. There are several pictures purporting to represent operations on the wounded, often with little artistic success. "Human Sacrifice" (212) is one of the worst of these, as well as having much too melodramatic a title. Mr. Muirhead Bone's medical subjects are also disappointing: he has such excellent work, and such quantities of it, on the war at large, that the conclusion suggests itself that he found the hospital atmosphere uncongenial, or else that he never stayed long enough to breathe in it freely. Several of the medical pictures suggest the same idea: one gets the feeling that the artist ought to have lived in a hospital, a dressing-station, or what not, for a week or two before he began to paint.

### The Best Pictures.

One of the R.A.M.C. pictures to which this criticism does not apply is that by Mr. G. Rogers (380), representing a stretcher party, which is altogether admirable. In the same gallery are quite a number of the best pictures of the collection, notably one by Mr. Hughes Stanton of the St. Quentin Canal; a lovely view of Harwich, by Mr. Charles Pears; a fine portrait of the celebrated airman McCudden, which is far superior to that of his equally heroic comrade Ball; and another masterpiece by Mr. Stanton (400), showing the cemetery at Mount St. Eloi.

Taking the exhibition as a whole, the military pictures by Mr. E. Kennington, and the naval pictures by Mr. Pears, maintain the most consistent level of excellence. The former has two casualty clearing-station themes, but the latter avoids medical subjects altogether. Incidentally Mr. Kennington is the only artist courageous enough to illustrate the "plague of lice"; so far as we could see no one at all depicted the equally ubiquitous trench rat. When the collection reappears at the Crystal Palace its attractiveness will have been enhanced, it may be hoped, by judicious elimination: at present there are too many canvases which simply serve by contrast to make one realise how very close to nature the Bairnsfather cartoons contrived to get.

